# Corticosteroids and immune checkpoint blockade

**DOI:** 10.18632/aging.100797

**Published:** 2015-08-17

**Authors:** Le Min, F. Stephen Hodi, Ursula B. Kaiser

**Affiliations:** Division of Endocrinology, Diabetes and Hypertension, Brigham and Women's Hospital, Boston, MA, 02115 USA

Novel immunotherapies acting through immune checkpoint blockade to stimulate the immune response have demonstrated promising outcomes in advanced malignancies [1]. As a result, novel cancer immunotherapy was named a scientific breakthrough of the year in 2013 by Science. Consistent with its mechanism of action of *de novo* stimulation or enhancement of pre-existing T cell responses, a number of immune-related adverse events (irAEs) have been observed in patients treated with ipilimumab.

Management of irAEs is challenging. The dilemma is that on one hand, the purpose of immune checkpoint inhibition is to stimulate the immune response against cancer cells [1]; on the other hand, such therapy can also induce autoimmune side effects against normal cells and tissues. Treatment of immune checkpoint inhibition-related autoimmune disorders with high dose corticosteroids (HDS), which is the usual therapy for autoimmune diseases, would be expected to result in immunosuppressive effects and paradoxically may counteract the anti-cancer activities induced by immune checkpoint blockade. The available data on the effects of HDS therapy on the outcome of anti-tumor activity from animal and clinical studies are limited and mixed. In an animal study [2), high dose dexamethasone given 7 days after anti-CTLA4 treatment blunted the anti-cancer effect of CTLA4 inhibition. More importantly, concurrent treatment with anti-CTLA4 and dexa-methasone ultimately resulted in reversal of tumor growth inhibition. A study by Downey *et al.* [3] to evaluate the anticancer activity by CTLA4 blockade in melanoma patients did not show a statistically significant difference in survival in responders with and without HDS treatment; however, the authors reported that, “Twelve patients were treated with steroids with a median duration of response of 19.3 months, which was somewhat less than the median response for all responders of 30.6 months. The 11 responders not treated with steroids have not yet reached a median duration of response.” This finding suggests that steroid treatment may have resulted in a shorter duration of response. In contrast, a study by Weber et al. [4] showed that budesonide did not affect the overall tumor response. However, since budesonide given orally is largely degraded by the liver before it enters the systemic circulation, this therapy may have minimized systemic effects on tumor response. Therefore, one cannot conclude from this study that high dose systemic corticosteroids did not impact tumor response.

Sporadic idiopathic lymphocytic hypophysitis is a rare autoimmune disease, occurring at a rate of 6 cases per million per year, with a higher incidence observed in females than in males. Through unclear mechanisms, hypophysitis has emerged as one of the most common adverse events related to CTLA4 blockade, with an incidence ranging from 1% to 6% in early studies [4]. A consistently higher incidence of anti-CTLA4-associated hypophysitis (anti-CTLA4-H) was documented in more recent studies [5], suggesting an increasing awareness of this historically rare autoimmune disease. The diagnosis of anti-CTLA4-H is based on its typical manifestations, including pituitary enlargement (usually mild) with pan- or isolated anterior pituitary hormone deficiency. The general guideline for the management of immune checkpoint inhibition-related hypophysitis is HDS and hormone replacement. Because of the relatively benign course of anti-CTLA4-H and concerns about the potential counteracting effects of HDS on the antitumor activity induced by immune checkpoint inhibition, we performed a study to evaluate the outcome of anti-CTLA4-H in patients treated with HDS or with replacement doses of corticosteroids for their hypophysitis-associated secondary adrenal insufficiency (equivalent to 30 mg hydrocortisone or less). In this retrospective study, we did not find a significant difference in the outcome of anti-CTLA4-H in terms of duration or frequency of resolution of pituitary enlargement or of anterior pituitary hormone deficiencies between the groups treated with or without HDS [5]. Moreover, a trend towards higher mortality was observed in the group treated with high dose corticosteroids, albeit without reaching statistical significance [5].

Unlike anti-CTLA4 therapy, anti-PD1 therapy is more commonly associated with thyroid disorders than hypophysitis [6]. In a recent retrospective cohort study, anti-PD1-associated thyroid disorders most commonly present as painless thyroiditis with either a transient thyrotoxic phase followed by a hypothyroid phase, or as hypothyroidism without a preceding thyrotoxic phase [7]. The recommended management for the thyrotoxic phase is with beta-blockers, without high dose corticosteroids [7].

No definitive conclusions can be drawn from available data for outcomes of HDS treatment in terms of impact on resolution of irAEs or the impact on tumor response. A well-designed prospective randomized clinical trial is required to clarify these important issues. Until then, based on the results of available studies, we recommend initiation of hormone replacement and symptomatic management of most patients with immune checkpoint inhibition-related endocrinopathies without HDS. However, if there are symptoms or signs of adrenal crisis (i.e., severe dehydration, hypotension, shock), significantly abnormal biochemistry results such as severe hyponatremia, or severe thyrotoxicosis in elderly patients or patients with cardiovascular disease, HDS should be immediately initiated.
